# Medical Surveillance, Continuous Health Promotion and a Participatory Intervention in a Small Company

**DOI:** 10.3390/ijerph15040662

**Published:** 2018-04-02

**Authors:** Nicola Magnavita

**Affiliations:** Institute of Public Health, Università Cattolica del Sacro Cuore, Roma, Italy; nicolamagnavita@gmail.com; Tel.: +39-347-330-0367

**Keywords:** workplace, health promotion, work-related stress, anxiety, depression, participatory ergonomics, wellbeing, organisational development, medical surveillance

## Abstract

The workplace is an ideal setting for health promotion. The regular medical examination of workers enables us to screen for numerous diseases, spread good practices and correct lifestyles, and obtain a favourable risk/benefit ratio. The continuous monitoring of the level of workers’ wellbeing using a holistic approach during medical surveillance enables us to promptly identify problems in work organisation and the company climate. Problems of this kind can be adequately managed by using a participatory approach. The aim of this paper is twofold: to signal this way of proceeding with medical surveillance, and to describe an organisational development intervention. Participatory groups were used to improve occupational life in a small company. After intervention we observed a reduction in levels of perceived occupational stress measured with the Effort/Reward Imbalance questionnaire, and an improvement in psychological wellbeing assessed by means of the Goldberg Anxiety/Depression scale. Although the limited size of the sample and the lack of a control group call for a cautious evaluation of this study, the participatory strategy proved to be a useful tool due to its cost-effectiveness.

## 1. Introduction

The workplace is an ideal setting for health promotion. In many European countries, including Italy, the employer is compelled to set up a health surveillance service for employees exposed to occupational health risks. This mandatory medical examination offers a valuable opportunity for gathering information on the health and wellbeing of workers. The occupational physician is able to monitor health and promote healthy lifestyles, thus transforming a preventive activity applied to aspects related to work only into a continuous program of general health protection and promotion. The transition from an activity that focused exclusively on the prevention of occupational diseases toward a strong commitment to health promotion is a natural evolution for occupational medicine that originated when levels of pollution in the workplace were much higher than today and social conditions were very different from the current ones. Nowadays psychosocial risk factors are of prime importance in occupational health [1,2,3,4,5], and require physicians to take a “holistic” rather than a “laboristic” approach to occupational health services in order to deal with these problems in the best possible way [6,7,8].

Effective workplace health promotion programmes require a strong commitment on the part of employers, managers and workers, as well as considerable medical staff involvement. The development of promotion campaigns that go beyond the usual health and safety activities in the workplace also requires specific funding and the acquisition of specialised skills. This is why it is fundamental that health promotion becomes part of the surveillance activity regularly provided for workers. Screening for general disease in the workplace makes possible secondary (early identification) or tertiary management (treatment) of health problems. It is also an occasion to spread best practices and correct lifestyles among workers (primary health promotion). In countries that have a national health service (NHS), workplace activities must be connected with it. Occupational health and safety (OHS) services that adopt this strategy usually establish each year the objectives of the promotion campaign in a participatory way with employers and workers. This work method enables them to identify a specific theme for promotion each time and to focus attention on workers at risk. 

A good rule of workplace screening is to measure psychosocial factors that can change the expression of the disease. This allows the physician to take into account occupational stress, which can be a significant factor confounding the relationship between environment and symptoms [9]. Contemporaneously, continuous screening activities that take into account the level of stress perceived by each worker make it possible to monitor the conditions of wellbeing and mental health in the workplace, and enable preventive organisational measures to be promptly implemented when necessary.

A promotion campaign that is part of routine medical surveillance in the workplace requires no additional investments other than those already allocated for occupational risk prevention. It should have a very flexible structure so as not to interfere with the ordinary activities of the health and safety service. The occupational health physician plays a leading role in this activity. He/she encourages other parties involved in health promotion to carry out risk assessment, medical surveillance, information and training, improvement actions and verification of results that are part of the virtuous risk management circle, according to the so-called ASIA© method [10,11]. The occupational physician plays a fundamental role in this process, especially in small companies, where resources and knowledge are often limited. This approach to workplace health promotion has been successfully applied in some companies since the early 1990s [12,13], but has been more widely used in the last 20 years.

Promotion campaigns included in routine surveillance involve administering a questionnaire containing three sections during medical examination: the first section concerns symptoms and early signs that may lead to diagnosis of the problem under study; the second analyses the main factors that can play a moderating role, for example work stress; the third deals with the consequences for physical and mental health. 

An analysis of these questionnaires yields two results: the identification of people at risk and health monitoring of the group. High-risk subjects identified during screening are invited to carry out further tests under the NHS and, if necessary, to undertake specific treatment. In general, the campaigns provide a detailed and repeated measurement of symptoms and complaints related to the working environment. The continuous search for improvement and health promotion incorporates prevention because a reduction in workers’ wellbeing can be promptly identified before the appearance of occupational diseases and specific action can be taken.

The health risks that emerge during health promotion campaigns can be addressed in different ways. If the risk factors are non-occupational, the physician will suggest ways of improving lifestyles and behaviours that workers may or may not decide to follow. If the survey highlights the presence of occupational factors, the employer must prepare a risk reduction plan. 

It can happen that medical surveillance highlights organisational problems. In these cases, it is desirable to implement organisational development strategies to assess work stress in the workplace [14]. Actions of this kind are primary interventions, focused on preventing stressors from even presenting, by defining employees’ roles, redesigning workflows, and increasing resources. It is advisable to involve the workers themselves in these preventive measures since they are the ones who identify the problem, suggest possible solutions and collaborate in their application.

Participatory action, i.e., a context-specific collective intervention wherein, through group discussions, workers help to identify problems in organisational structure, work processes and practices, as well as help to design, implement, and evaluate success of solutions, is often proposed in the literature. A meta-analysis of stress management interventions showed that organisation-level interventions proved to be effective at both the individual and organisational level [15]. A well-known example of participatory action research is the experience of Health Circles in Germany [16]. Bottom-up organisational interventions were also applied in Thailand [17], Japan [18], the Netherlands [19], and Korea [20]. In Canada a participatory organisational action obtained a significant increase in Reward and a significant decrease in Effort/Reward Imbalance in health care workers [21].

The aim of this paper is twofold: to signal this new way of proceeding with medical surveillance, which is typical of modern occupational health, and to describe a specific organisational intervention.

In a small company based in Rome, Italy, a gradual decline was observed in the level of the workers’ psychological wellbeing during health promotion campaigns. This was confirmed by interviews conducted during medical examinations and prompted specific action designed to identify the causes and suggest solutions for improving the quality of working life. This intervention was carried out in 2016 by means of participatory ergonomics groups (GEPs). This article reports the observations and the short-term results of that intervention.

## 2. Materials and Methods 

### 2.1. Population

The company in question is a welfare and assistance agency for professionals. Since 1997 its employees have undergone mandatory health surveillance, mainly due to the “video terminal” occupational risk. Italian law stipulates that workers assigned to video terminals must undergo regular medical examination to assess their fitness for work. With the consent of the workers and the employer, in this company medical check-ups were also used for health promotion purposes. Over the years, promotional activities focused on numerous topics: indoor air quality, musculoskeletal disorders, workplace violence, aging, sleep and fatigue, and others, following a schedule agreed with the employer and the workers. 

The company’s offices employ 57 people, with a slight prevalence of female gender. Due to the low turnover, the population has remained almost constant in number and has aged progressively. In 2015 the mean age was 47.4 (+4.9) years, with 26 male and 31 female employees; in 2017 one male worker left the company, and one female worker was taken on. This led to a slight change in the aforementioned parameters (age 49.0 + 5.8 years, 25 M, 32 F).

### 2.2. The Technique of Participatory Groups

Primary stress prevention intervention, through reorganisation of work, was carried out through participatory ergonomic groups.

Setting up participatory ergonomic groups (“Gruppo di Ergonomia Partecipativa”, GEP©) is a way of encouraging worker participation in improving working conditions by means of a bottom-up approach. The GEP© method is based on meetings during which all the workers who contribute to the performance of a specific work task describe their working activities in detail and identify any critical aspects. Once a problem has been identified, workers are urged to seek and discuss solutions to the problem and choose the one that appears to be the most economical and feasible. This solution is then formally presented to the management for analysis and implementation. The effectiveness of this group activity primarily depends on the ability of its members to interact with each other and find an agreement. The GEP© therefore has the function of increasing the ability to collaborate within the group and to seek collective and not individual solutions. 

GEPs© were initially developed in the industrial field to solve safety problems [22] or to improve the quality of production. Subsequently, this method was applied to services, and was used to improve production processes and work organisation. The application of GEPs© for the prevention of musculoskeletal disorders in hospitals was given appraisal in 2007 in the European “Lighten the load” campaign [23] and was given an award in 2008 by the Italian Society of Ergonomics [24]. GEPs© were also used for the promotion of behaviours that prevent aggression against staff in the workplace during a complex multi-year program [25] that was included in OSHA’s Guidelines for Preventing Workplace Violence for Healthcare and Social Service Workers [26]. In 2017, the application of GEPs© to the health promotion of older workers was given an award in the European ‘Healthy Workplaces for All Ages’ Campaign 2016–2017 [27,28]. 

Unlike structured focus groups, where the moderators need to be visible and take an active role in the group and participants are likely to answer the set questions posed by the moderators [29], in GEPs there are not many questions, but only one: the work that the unit has to do. The doctor primarily aims to facilitate the discussion on this topic, and the workers are encouraged to talk to each other.

The first meeting begins by inviting one of the workers to describe the work cycle and the other workers to intervene by detailing the operations that are done. The workers in turn identify the problems that arise within the work cycle and those that originate in relation to the other processes of the company. Workers speak about the problems identified, not laying blame, but focusing on the causes. They are therefore invited to propose solutions for each of the identified causes. In the various meetings the workers discuss the causes and solutions and choose the simplest, most acceptable and most economical solutions, which are then proposed to the management. The technique of GEPs, which was initially developed to prevent injuries and trauma, was gradually extended to deal with organisational problems. To assess work-related stress, the observations of workers participating in the GEP© are classified on a grid that includes the three most common complementary models of work-related stress. The Karasek model [30] includes the Demand/Control/Support variables. According to this model, “high job strain” can occur in cases of excessive psychological work burden (“demand”), with insufficient “Control” over the job. In this model, social support is an important moderator of stress. Siegrist’s model [31] postulates that excessive stress occurs when the effort made by the worker (“Effort”) does not meet with adequate rewards (“Reward”); the intrinsic component of the model (“Overcommitment”) may have an interactive or independent role [32]. The third model refers to organisational justice [33,34], which is divided into procedural, distributive, interpersonal and informative justice. In GEPs, the problems reported by workers are therefore classified into 10 categories of factors related to occupational wellbeing (demand, control, support, effort, reward, overcommitment, procedural justice, distributive, interpersonal, and informative justice). This classification, made by the doctor, has the primary purpose of describing and rationalising the arguments. In fact, the doctor is not a psychologist and does not intervene on individual efforts to master demands or coping strategies, nor treat harmful repercussions of stressors that were not coped with sufficiently. The group approach is the search for the best way to work. In large companies the task of giving voice to the people in the design of interventions to improve work organisations is under the supervision of a professional in psychosocial risk prevention and management. In SMEs the occupational doctor works together with managers and employees to find the best collective solution. One or more solutions can be proposed by the GEPs for each of the observed problems.

Although the occupational health physician promotes the GEP© meetings, he/she plays no active part in them: he/she merely records the problems reported, compares them with the main models of work-related stress to verify the existence of risk conditions, and reports the proposals for improvements formulated by the workers. These proposals are then evaluated by the company, which decides whether or not they are applicable.

### 2.3. Questionnaires and Participatory Groups

In this company we identified six work groups engaged in the same number of occupational tasks. Each group, composed of all the workers (managers, officials, employees and clerks) who were involved in carrying out a specific operational task, met in the presence of a physician to describe their work, indicate the key issues and develop and discuss possible solutions. The GEPs were set up in 2016 and all the workers participated. The proposals developed by the GEPs were then transmitted to the company managers, who proceeded with their gradual application during 2017.

The workers underwent medical examinations in 2015 and 2017. On these occasions they participated in two different health promotion campaigns: the first concerned aging of the workforce, chronic diseases and work ability; the second aimed at promoting sleep health and counteracting metabolic syndrome. On both occasions, work-related stress and psychological wellbeing were measured. For the purposes of this specific study, we will compare the data collected in 2015, prior to the establishment of GEPs, with those of 2017, i.e., after implementation of the improvements suggested by the workers.

Some questionnaires were used for both health promotion campaigns conducted in 2015 and 2017. These questionnaires measured work-related stress and the risk of developing minor psychiatric disorders (anxiety and depression), respectively.

Work-related stress was measured in 2015 and 2017 with the ERI questionnaire (the short, validated version in Italian) [35]. The questionnaire consists of three items for Effort, seven for Reward and six for Overcommitment. ERI is calculated as a weighted ratio between Effort and Reward; values above the unit indicate an imbalance between Effort and Reward. The reliability of the scales of the questionnaire in this research, measured by Cronbach alpha, was 0.77 for effort, 0.80 for reward and 0.65 for Overcommitment.

The measure of mental wellbeing used at baseline and at follow-up was Goldberg’s Anxiety and Depression scale [36], a simple list of 9 + 9 binary items initially designed to enable General Practitioners to identify which of their patients may be at risk of developing mental disorders. A person who reports a score of over 4 on the scale of anxiety and more than 3 on the depression scale has a 50% chance of developing a pathological condition, and the risk rises rapidly with the score. In this survey, the questionnaire was found to have a good reliability (Cronbach alpha = 0.88 for anxiety, 0.79 for depression).

During the medical visits in 2017 the workers who had participated in the GEPs were individually questioned about the results of the organisational intervention. Given the very limited time, workers’ answers may be seen as a guess rather than data supported by evidence. However, they represent a process evaluation [37] documenting program functioning, and identifying barriers to implementation [38].

This study was approved by the Ethics Committee of the Università Cattolica del Sacro Cuore of Rome, ref. n. 139/2016; 02/2010.

### 2.4. Statistics

The scores of work-related stress and those of anxiety and depression at baseline and at follow-up were compared with the Wilcoxon U test for paired data. The analysis was performed with IBM SPSS© Statistics 23.0 (IBM Corporation, Armonk, NY, USA).

## 3. Results

The six groups were made up of all the workers who were part of one of the six sections in which the company’s work is articulated. The participants in each group were from six to 15 people. Each group met on average four times to identify problems and propose solutions. Both the problems and the solutions proposed emerged and were validated in all groups and by all workers.

The observations formulated during the GEPs© were initially analysed using a grid composed of the 10 variables that contribute to determining work-related stress.

An analytical examination of the results shows that all stress-related variables are mentioned at least once in each of the groups of workers consulted. Table 1 summarises the observations made in the GEPs© with reference to the different stress-related variables. 

In the six different offices in which GEPs were formed, the workers agreed to describe the same problems related to the work climate. Interpersonal relationships appeared to be difficult on account of the excessively authoritarian style adopted by the managers. This attitude was determined and aggravated by a constant lack of time, tight deadlines and limited human and material resources available for carrying out the necessary amount of work. In this climate of general tension, the relationships between different offices of the same company were also problematic, partly due to an attempt to offload their own inefficiencies onto others, and partly on account of a reciprocal failure to recognise the work performed by each operating unit. The latter, i.e., the lack of material and immaterial rewards for the work done, was most frequently reported as a cause of work dissatisfaction. More generally, some workers reported a lack of confidence in the company’s prospects and in the management’s desire to guarantee a future for the enterprise.

The workers proposed an intervention for each of the main problems detected in work organisation. Some of these were discarded as being too expensive or difficult to apply; others were rejected on the grounds that the intervention would lead to unpredictable results or consequences that would not obtain general approval. 

The solutions that appeared simpler and cheaper and that obtained universal approval are shown in Table 2. These solutions were presented to the managers, who gradually implemented them in 2017. Improvement is still underway.

In 2017, the company modified the workload for some managers by reducing their responsibilities and by setting out the tasks of each operating unit in greater detail. In all the operating units, regular meetings were introduced to schedule the weekly work commitment and solve any critical issues. Action designed to increase the recognition given to workers was planned at various levels: e.g., the promotion of some employees to higher functions; the establishment of a reward system for the most active employees; public recognition of successful achievements at work. A policy against violence at work was also introduced, with punishments for verbal aggression. Furthermore, a decision was taken to promote convivial group activities, outside working hours, to allow short breaks for relaxation during the working day, and to broadcast background music in the workplace.

During the visits made in 2017 the workers mainly expressed very moderate satisfaction with the intervention. The prevailing opinion was that the changes adopted by the company had been too slow and partial. Some have complained that there was no way to counteract problems outside the company, which lead to tension in workers. Others have reported a lack of interventions in terms of interpersonal relationships. The workers, however, appreciated that, through the GEPs, the problem of the organisation of work had been brought to the attention of the management.

A comparison of the levels of work stress perceived before this intervention on the part of the GEPs and those recorded in 2017 showed a slight, but significant increase in the mean score for Rewards and a decrease in the mean score for Effort. Overcommitment remained substantially unchanged. Anxiety levels showed a reduction that failed to reach the level of significance, while the depression score was significantly reduced (Table 3).

## 4. Discussion

In this study we observed that the changes in work organisation that the company introduced in order to comply with indications from the groups of workers resulted in a slight, but significant, reduction in work-related stress. In particular, a reduction in perceived Effort and an increase in Reward were observed, while Overcommitment, or intrinsic stress, remained substantially unchanged. This result is in line with expectations, as the intrinsic component, or coping pattern, is more stable than the extrinsic components of the stress model [32]. Following the intervention on work organisation, we also observed a significant reduction in the mean depression score and a non-significant reduction in the anxiety score.

Our study demonstrates that medical surveillance in the workplace can lead to a positive continuous health promotion activity, thereby encouraging healthy lifestyles and correct health practices in the population. Using a broader salutogenic approach for the continuous monitoring of workers’ health is more beneficial than the mere prevention of occupational diseases, as it can detect the appearance of crucial issues in work organisation before the latter induce health disorders. 

When medical observation shows the presence of occupational stressors, it is appropriate to plan specific interventions, such as the one examined in this paper. GEPs© can be a useful tool for revealing problems in work organisation and for formulating joint solutions. Our findings indicate that the changes introduced in a small company were associated with an improvement of the working climate by reducing stress levels and increasing the level of psychological wellbeing of the workers. This approach strives to attain synergistic effects by coordinating activities carried out at multiple levels. By integrating the periodic medical examinations that the law prescribes to control professional risks (such as work with video terminals) with the screening of disturbances that do not originate from the work but can alter work ability, OHS services make secondary and tertiary promotion. With this continuous activity of promotion and monitoring the levels of wellbeing and occupational stress, it is possible to test organisational interventions for the primary prevention of psychosocial risks. The main result of this study was the proposal of an approach that can be successfully applied to occupational health promotion in all workplaces. The succession of annual secondary/tertiary health promotion activities makes it impossible to understand which part of the improvement observed is due to the primary promotion intervention carried out with the GEPs, and which part derives from the annual campaigns. This question is relevant from the scientific point of view, and only subsequent multicentric studies with internal control groups, in which only one of the two types of intervention is conducted at a time, can solve the question. However, from the point of view of the occupational physician of a small company, the most important result is that, with these actions, worker health has improved.

The intervention described is specifically designed for small and medium-sized enterprises (SMEs), which in Italy represent 99.9% of total companies. While large companies often have a staff that includes various professionals involved in the prevention of work stress, in small and medium companies (SMEs) there is only one doctor. Faced with a choice between doing nothing at all and the doctor making a low-cost intervention, this company decided in favour of the latter.

The intervention of the occupational doctor through the GEPs is an approach to the problem of work stress that is very different from what would be possible through industrial and organisational psychology. In particular, the intervention through GEPs cannot modify some aspects of the working environment that are relevant to mental health, such as individual resources, organisational hierarchy, leadership styles and interpersonal relationships. This last aspect was particularly critical in the company in question, given that the workers have spent many years developing friendships and very often marriage relationships. The relational complexity transcends the scope of occupational health. Based on this experience, we propose implementing in the near future in this small company an organisational psychology action enhancing resilience and improving managerial style and interpersonal relationships.

Health promotion is based on the principle of making people better able to control and improve their health. The workplace offers an ideal opportunity for health promotion activities. In this type of setting, a participatory approach to intervention for improving the work environment yields optimal results. This particular type of occupational health setting is referred to as the “supersetting approach” [39]. 

An approach similar to the one we adopted can be found in studies such as the Australian Healthy Workers Initiative, which takes an embedded approach to workplace health promotion [40], or the Center for the Promotion of Health in the New England Workplace, an intervention research study that combines ergonomics with health promotion [41]. In the USA, the NIOSH Total Worker Health (TWH) explicitly recognises that the health and wellbeing of workers are conditioned by the work environment and extra-work activities [42]. In small and medium-sized enterprises in the USA [43], India [44] and Australia [45], other workplace interventions combining health protection and health promotion were conducted to create a workplace culture that valued and efficiently managed health, safety and wellbeing, with a special emphasis on organisational productivity. A recent review of the literature gives some support to the idea that integrated interventions targeting occupational health and safety management may offer comprehensive solutions to complex workplace issues [46]. This type of approach seems to be advantageous, especially for mental health problems [47]. On the contrary, promotion programmes aimed exclusively at modifying behaviours and educating individual workers without transforming the workplace culture may fail to obtain significant effects [48].

Occupational health activities in the workplace have two objectives: one is to protect the health of the individual worker and can be assessed in terms of risk/benefit ratio, while the other is an epidemiological aim designed to improve the health of the group, and can be evaluated in terms of cost-effectiveness. Our intervention had a very favourable risk/benefit ratio, because the risk for a worker in adhering to a promotion campaign and filling in a questionnaire is negligible compared to the advantages resulting from diagnosis of an illness or a condition of high risk. Similarly, at a group level, annual health promotion campaigns have an extremely favourable cost/effectiveness ratio, as the cost of a statistical analysis of the data collected through questionnaires is minimal considering the epidemiological results collected. Using GEPs to intervene in problems concerning the working environment also has an excellent cost-effectiveness ratio, since the effectiveness of the safety measures developed with the help of the workers more than compensates for the time the physician and employees need to identify crucial issues and propose solutions. Worker involvement increases the efficiency of the ergonomic process and enables the resources needed for promotion and prevention interventions to be used in the best possible way. The GEP© strategy takes time, but requires above all a strong desire for collaboration on the part of both workers and the management. If this willingness to cooperate is lacking or weakens, and conflict or a search for personal solutions prevails, this kind of strategy may not be as effective as in our study.

Despite the aforementioned positive aspects, this research study has some limitations. The small size of the company, which enabled us to implement a low-cost programme, constituted the principal limitation of the study. In a small sample there is clearly great variability and the final results can be influenced by a few units. Moreover, the observation interval was not large enough to assume that the improvement observed was stable and irreversible. Further efforts are needed to develop new solutions and verify their effectiveness. The GEP© strategy can also be of help in providing this corroboration. 

In this paper an evolving situation has been described. In fact, it is necessary to observe that only some of the proposals made by the GEPs were welcomed by the management and that the implementation of some accepted proposals is also underway. On the other hand, both working methods and health promotion/risk prevention are constantly evolving in all work environments.

In the case in question, two types of promotional actions were carried out at the same time: cross-sectional screenings and organisational intervention. It is not possible to know which part of the observed improvement is to be attributed to the screenings and which to the reorganisation.

A limitation common to most of the studies conducted in small companies is the lack of a control group. For ethical reasons it is not possible to exclude some of the workers from an organisational improvement. We hope that after this pilot study a comparison between parts of a company in which the reorganisation of work through the GEPs has already been done and unmodified control departments can take place in large companies. Another limitation of the study is that, in order to verify the results of the solutions suggested by the GEPs, we used a single model of stress, the effort/reward imbalance model. We must take into account the fact that the results might have been different if we had used the demand/control/support model or that of organisational justice. However, since all three complementary models of work-related stress have previously been used in the small company to measure self-perceived stress, in the future it will be possible to verify the effect of the actions taken by applying the other stress models. We must remember that the use of more advanced stress models (e.g., cybernetic model, demands–resources) [49] remains the prerogative of occupational psychology interventions. Another limitation is that in this study we used questionnaires, a subjective measure, rather than objective measures such as sick leave or productivity indicators. Future research could compare the subjective evaluation of workers with objective indicators, although it should be remembered that self-assessment questionnaires have always proven to be a valid measure of workers’ stress and health status.

## 5. Conclusions

In conclusion, we are convinced that using health surveillance in the workplace to carry out continuous health promotion campaigns is better than the traditional approach, which focuses only on the prevention of occupational diseases. GEPs© can be a useful tool for addressing the health risks that originate within the workplace. The participation of workers in seeking solutions to improve their occupational life increases their commitment and work engagement. The literature is unanimous in recognising that work engagement is linked to job satisfaction, productivity, and the health and safety of workers. This should urge companies to apply strategies such as GEPs for improving worker wellbeing and the quality of work.

## Figures and Tables

**Table 1:** Work-related problems identified by workers.

Variable	Type of Problem
DEMAND	Increase in work due to changes in rules and laws. Workload is sometimes excessive, and unevenly distributed. Extension of work beyond office hours.
CONTROL	Difficulty updating skills. Excessive concentration of responsibility in the hands of a few people, lack of proxies.
SUPPORT	Gradual deterioration of relationships.
EFFORT	Fatigue derives more from relationship problems between people or groups than from the work to be carried out.
REWARD	No recognition for the work performed or encouragement in the event of difficulties.
OVERCOMMITMENT	Some workers are busy at work 24 h a day.
PROCEDURAL JUSTICE	Procedures are not always verified.
DISTRIBUTIVE JUSTICE	Inequalities in responsibility loads. Uncertainty about individual responsibility for tasks.
INTERPERSONAL JUSTICE	Significant lack of correct conduct in relationships, verbal violence.
INFORMATIONAL JUSTICE	Lack of information. Excessive gap between managers and other workers.

**Table 2:** Solutions proposed by workers that have been implemented (I), are in progress (P), or will be considered in the future (C) by the management.

Variable	Solutions
DEMAND	Check the workload assigned to the different offices and individuals (P). Establish a system of verification of the activity carried out (C). Define the correct use of emails (I). Organise operational meetings with external experts in order to solve problems (C).
CONTROL	Improve training, not only on specific topics, but also on how to communicate (C). Assign specific responsibilities (I). Share corporate objectives and projects (P).
SUPPORT	Organise convivial occasions. In addition to the methods used in the past (parties, social outings, etc.), introduce collaborative activities, meetings in which everyone produces something) (I). Stimulate hidden abilities—hobbies, workers’ activities—with a reward system (C).
EFFORT	Training courses to increase resilience (C). Summer camps for employees’ children (C). Establishment of moments of relaxation in order not to accumulate tension that is then taken out on colleagues (P). Background music in the workplace (I).
REWARD	Establish a reward system (P). Formal recognition (not necessarily pecuniary) that matches the objectives achieved with the means available rather than with needs (P). Awards for workers who improve collaboration within the company (P). Pecuniary recognition of functions (I)
OVERCOMMITMENT	Counter the habit of sending emails or phone calls outside office hours (I).
PROCEDURAL JUSTICE	Verification of procedures (I). Planning of company’s activities (C). Planning of work in the different offices and their relations (P). Obtain feedback from operators on how the programmes work (I). Establish the cause of errors, not those responsible for errors (I). Share decisions about training methods, software purchases, etc. (I)
DISTRIBUTIVE JUSTICE	Establish a system of authorising and assigning responsibility. (I) Time commitments correctly, avoid delays or inefficiencies in other offices. (I) Avoid concentrating tasks in the hands of a single person. (I) Reassessment of tasks and assessment of productivity. Match pay with responsibilities. (I) Turnover between controllers and the controlled. (C)
INTERPERSONAL JUSTICE	Code of conduct, in the relationships between individuals and between offices. (I) Policy against verbal violence. (I)
INFORMATIONAL JUSTICE	Regular work meetings to be carried out in each operative unit. (I) Regular exchange of information between offices on procedures and activities produced. (I) Production of an internal newsletter. (C)

**Table 3:** Characteristics of the population. Levels of occupational stress and of anxiety and depression risk.

Variable	Baseline	Follow-Up	Difference (s.d.)	p ^(^*^)^
Age, years	47.4 (±4.9)	49.0 ± 5.8		
Population	57	57		
Male, *N* (%)	25	24		
Effort	9.27 (1.82)	8.73 (1.82)	−0.545 (1.40)	0.007
Reward	14.98 (2.54)	16.56 (3.97)	1.582 (3.95)	0.004
ERI	1.48 (0.37)	1.34 (0.60)	−0.138 (0.55)	0.002
Overcommitment	16.09 (3.69)	15.29 (2.66)	−0.800 (4.88)	0.261
Anxiety scale	6.13 (2.49)	5.67 (2.87)	−0.455 (2.20)	0.077
Depression scale	4.44 (2.61)	3.91 (2.47)	−0.527 (1.75)	0.025

^(^*^)^ tested with the Wilcoxon U test for paired data.
